# The effect of asymmetric reproductive ability on the evolution of cooperation on interdependent networks

**DOI:** 10.1038/s41598-019-46826-0

**Published:** 2019-07-24

**Authors:** Chunpeng Du, Yini Geng, Xiaoxiao Yin, Yongjuan Ma, Xiaogang Li, Lei Shi

**Affiliations:** 1 School of Statistics and Mathematics, Yunnan University of Finance and Economics, Kunming, Yunnan 650221 China; 2 Zhejiang College, Shanghai University of Finance and Economics, Jinhua, 321013 China; 3 School of Statistics and Mathematics, Shanghai Lixin University of Accounting and Finance, Shanghai, 201209 China

**Keywords:** Statistical physics, Complex networks

## Abstract

In this paper, we consider an asymmetric reproductive ability on interdependent networks and investigate how this setting affects the evolution of cooperation. In detail, players decide to update their strategies at each step on main network (network *B*), while for sub network (network *A*), players update their strategies with a fixed probability *p*. Obviously, the system restores the traditional case when *p* = 1, where cooperation can survive by interdependent network reciprocity. And our asymmetric set-up comes into play when *p* < 1. Numerical simulation results show that our asymmetric coupling will hinder the overall cooperation level for small *p*. In detail, the introduction of asymmetric reproductive ability urges the formation of symmetry breaking and further weakens the positive impact by location synchronous effect. And the root cause is entirely distinct situation of utility differences on two networks. These observations further demonstrate a class of phenomena on interdependent networks that it would have catastrophic consequences on one network even if a unrelated change only occurs seemingly on another network.

## Introduction

According to theory of natural selection^1–3^, Darwin^4,5^ presented the significance of competition on the evolution of species. However, it was found that cooperation also plays an essential role over the course of evolution and is widespread in both human society and ecosystems. Thus, exploring the emergence and maintenance of cooperation in this competitive society became a worthwhile challenge^6–9^. In previous work, Nowak^10,11^ summarized five rules for the evolution of cooperation named kin selection, direct reciprocity, indirect reciprocity, network reciprocity, and group selection in 2006. In particular, network reciprocity, cooperation can survive by forming compact clusters to resist the invasion of defectors, gave us a new perspective to address this overreaching problem. Along this framework, many social mechanisms have been considered, such as reputation^12–15^, social diversity^16,17^, punishment and reward^18–21^, memory^22^, co-evolution scenarios^23–26^, and so on^27–30^. For a comprehensive understanding, please refer to^31–33^. In addition, the progress of network science also played an important role in exploring the evolution of cooperation. The propose of new network topologies, such as coevolving network^34,35^, small-world^36^ and scale-free network^37^, *et al*.^38–41^ enriched the study of this field.

However, more realistically, people always locate on different networks simultaneously, which means behaviors on multiple networks codetermine the success of these people. Thus, the theory of interdependent networks was proposed and mechanisms such as probabilistic interconnectedness^42^, interdependent network reciprocity^43^, nontrivial organization of cooperators across the interdependent layers^44^ and information transmission^45^ was investigated gradually. From their studies, we found that cooperation can be facilitated under location synchronous effect. Furthermore, some other results showed that if two networks are interdependent, although a seemingly unrelated change occurs only on one network, it would have catastrophic consequences on another network^46–48^.

In general, evolutionary game theory has traditionally assumed that all individuals in a population interact with each other between reproduction events. However, it is found that there is time difference between the process of reproducing and interacting. Especially in the biological evolution^49–51^, selection often occurs much slower than that having interactions between individuals. Based on this phenomenon, a number of mechanisms were studied on a single layer network. Roca et^52^ proved that considering independent interaction and selection time scales leads to highly nontrivial and counterintuitive results. They also demonstrated that rapid selection may lead to changes of the asymptotically selected equilibria, changes of the basins of attraction of equilibria, or suppression of long-lived metastable equilibria. Besides, Rong *et al*.^53^ proposed an adaptive strategy-selection time scale model and showed that cooperation can be promoted if the learned information is properly used.

Although they made a great progress on considering time scale, these works are focus on single network. Thus, it is natural to investigate its performance on interdependent networks. In detail, we design an asymmetric coupling rule on interdependent networks and investigate how this affects the evolution of cooperation. Here, players on the main network (network *B*) decide to update their strategies at each step, while for players on the sub network (network *A*), they update their strategies according to a fixed probability *p*. The system restores to the traditional case when *p* = 1, where cooperation can survive by interdependent network reciprocity. Numerical simulation results show that our asymmetric coupling will hinder the overall cooperation level for small *p*. Interestingly, cooperation rate increases with the increasing of *p* for sub network, while cooperation decreases with the increasing of *p* on another network. The rest of this paper is organized as follows. First, we describe the public goods game and asymmetric reproductive ability on two networks. Second we introduce the main results, and finally we summarize and discuss the significance of this study.

## Results

**Figure 1:**
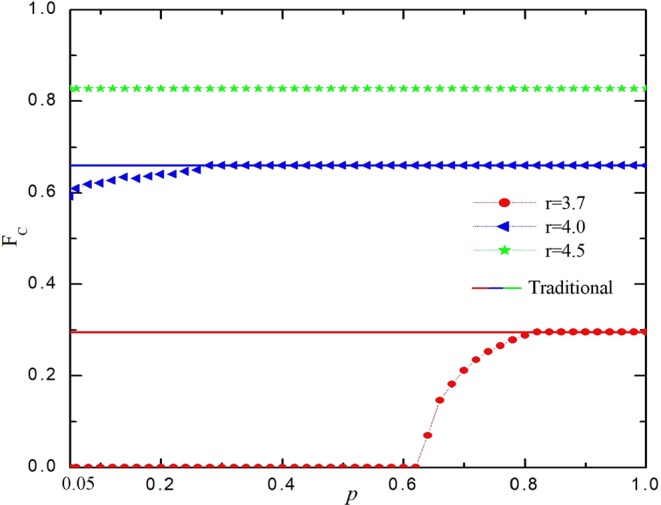
Asymmetric reproductive ability couldn’t provide an easeful environment for the evolution of cooperation. Fraction of average cooperation as a function of selection probability for different values of enhancement factor *r*. The open circle connected to the dotted line represents the traditional state (*p* = 1), the closed circles connected with a solid line represent the average cooperation level of the interdependent network, which with time scale attribute.

Above all, we first study how reproduction ability *p* affects the evolution of cooperation. Figure 1 features average fraction of cooperation on two networks in dependence on reproduction ability *p* for different values of enhancement factor *r*. We can find out that our setup couldn’t provide an easeful environment for the evolution of cooperation no matter which value of *r* is applied for relative small *p* compared with traditional case (solid lines), where cooperation can survive by interdependent network reciprocity^54^. Particularly, this negative effect fades away with increasing *p* until the cooperation level in line with that of traditional version. However, the cooperation level can be proved by location synchronous effect in interdependent networks for traditional case^55^. Thus, according to our speculation, the reason for this phenomenon is that the emergence of reproduction ability disrupts the location synchronous effect observed in interdependent network reciprocity. Furthermore, the negative impact of *p* becomes less obvious with increasing *r*. In order to get a comprehensive understanding about these results, in what follows, we then investigate the evolution of cooperation on both networks.

**Figure 2:**
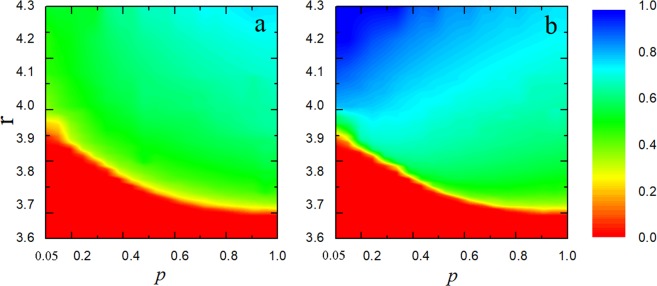
The cooperation levels of two networks appear to a far different situation. Colour-code (see bar on the right) fraction of cooperation on the *p*-*r* parameter for network *A* (**a**) and network *B* (**b**).

Figure 2 presents color maps encoding the fraction of cooperation ρ_c_ on the *p* - *r* parameter plane for two networks (network *A* and *B*). It is obvious that the larger value of *r* corresponds to the higher level of cooperation by means of strengthening network reciprocity no matter whether considering reproduction ability *p*. However, the cooperation levels of two networks still appear to a far different situation. Cooperators occupy an overwhelming majority of population when the value of *r* is pretty large on network *B* in Fig. 2(b), while for network *A* (Fig. 2(a)), cooperators and defectors are neck-and-neck. As we inferred above, the introduction of reproduction ability *p* only on network *A* could restrain the location synchronous effect. In this case, the phenomenon of two networks shows great asymmetry. And it is difficult for cooperators to strive for more vivosphere under the influence of symmetry breaking.

**Figure 3:**
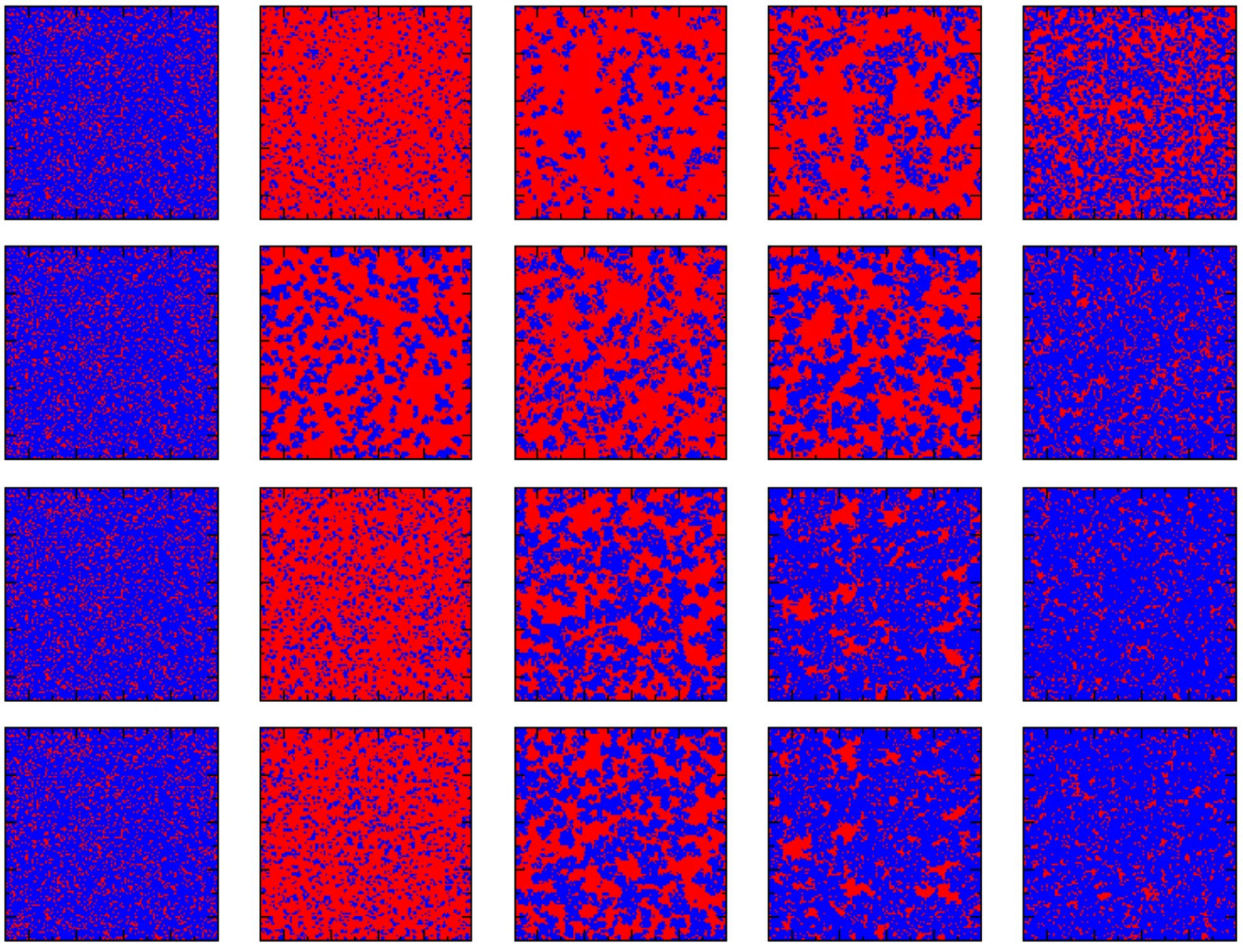
The asymmetric reproductive ability breaks the location synchronous effect. Snapshots of the distribution of cooperators (blue) and defectors (red) on the two interdependent square lattices at 0, 50, 200, 1000 and 50000 MCS from left to right. The above two lines represent the case of *p* = 0.05, and the first and second lines represent the network A and the network B, respectively. The bottom two lines represent the traditional case (*p* = 1), and the third and fourth lines represent network *A* and network *B*, respectively. Parameter values are r = 4.0, *L* = 400.

Next, in order to explore the results at the micro level, we give an evolutionary snapshot of strategies on network *A* and *B* for different values of *p*. As presented in Fig. 3, the top and bottom two lines of snapshots show the evolutionary process of cooperators (blue) and defectors (red) for *p* = 0.05 and *p* = 1 (traditional version) on two networks, respectively. Initially, cooperators and defectors are randomly distributed on square lattice for both situations. We first consider the traditional case (bottom two lines), where individuals on both networks decide to learn strategies from their neighbors by probability *p* = 1. Evidently, nearly the same processes of evolution take place on network *A* and *B*. Defectors can dominate rapidly by exploiting cooperators they connected with, and then lose ground for lower payoffs as the number of defectors grows. In this way, cooperators have opportunities to prevail by forming clusters. Namely, cooperators could behave well on both two networks ultimately under combined action of network reciprocity and location synchronous effect. However, the situations of two networks are totally different as numeration proceed for *p* = 0.05 (top two lines). In detail, cooperators on network *B* can survive and even prevail by forming enormous and tight clusters through network reciprocity. While for network *A*, slowing down the speed of evolution breaks the location synchronous effect with network *B*, which provides cooperators a less favorable environment. This symmetric result arises accordingly.

**Figure 4:**
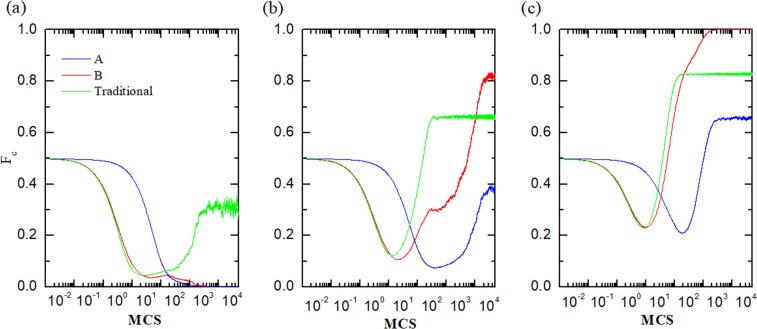
The enlargement of *r* could weaken social dilemma by shrinking the payoff differences between cooperators and defectors. Time courses depicting fraction of cooperation for network *A* and *B* (dotted line) and traditional case (solid line). Results are obtained for *r* = 3.7 (**a**), *r* = 4.0 (**b**), *r* = 4.5 (**c**).

To understand the role of the parameter *r* in the system, we further present the time evolution of cooperation on two networks for different values of *r*. Since the initial state is beneficial to defectors, the number of cooperators on two networks decreases rapidly at the beginning. And cooperators vanish in the end for pretty small value of enhancement factor *r* (Fig. 4(a)), while the frequencies of cooperators recover and even turn the tables when the value of *r* is large enough. Furthermore, cooperative behavior becomes prevail with increasing *r*. This is because the enlargement of *r* could weaken social dilemma by shrinking the payoff differences between cooperators and defectors. Besides, for the reason of influenced vastly by reproduction ability *p*, the cooperation level on network *A* is always lower than that of on network *B*, which corresponds to the results we obtained before. And the cooperation rates in traditional cases are between that on network *A* and *B*. We consider the direct cause of this phenomenon is utility differences.

**Figure 5:**
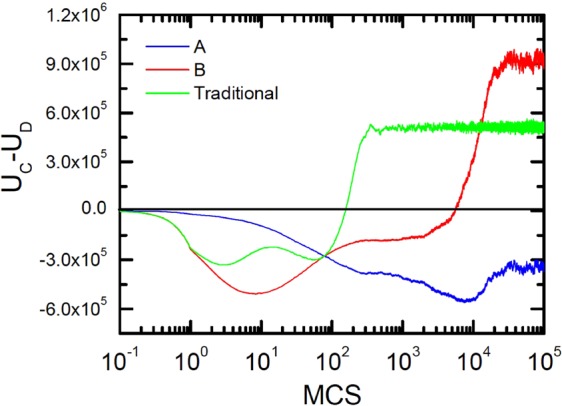
The introducing of reproduction ability *p* on network *A* slows down evolution of strategies, which makes defectors around the boundary have enough time to obtain more payoffs. Simultaneous time evolution of the differences in utility between cooperators and defectors on networks *A* (*p* = 0.05, blue) and *B* (*p* = 0.05, red) and traditional case (*p* = 1, green). Obtained for *r* = 4.0.

In order to verify our conjecture, we show the temporal evolution of the differences in utility between cooperators and defectors in Fig. 5. Like time evolution of cooperation in Fig. 3, the evolution of utility differences also experiences the feedback process of decreasing firstly and then increasing, while the time scale of these two evolutions are different. The same evolution trends of strategies always occur after the evolution of fitness, which means the updating of population depends heavily on utility and payoff. In fact, the introducing of reproduction ability *p* on network *A* slows down evolution of strategies, which makes defectors around the boundary have enough time to obtain more payoffs. Thus, it is difficult for cooperators to resist the erosion of defectors with higher payoffs. However, the same situation couldn’t happen on network *B*, where the circumstance of cooperators is even better than that of traditional version for higher utility difference between cooperators and defectors. This leads to the appearance of asymmetric structure directly.

## Conclusion and Discussion

In our work, we have proposed and analyzed an evolutionary public goods game including a selection probability *p* in a layer of network by interdependent networks. Our results show that asymmetric reproduction could hinder the evolution of cooperation for overall cooperation level, and the cooperation level between the two networks is vastly different, especially when *p* is small. The combination of these factors also gives rise to rich dynamic behavior of system. In particular, defectors on network *A* can exploit more benefits from cooperators in the neighbor through introducing of reproduction probability *p*, where the frequency of updating strategies is slowed down. However, players on network *B* without *p* behave totally different. In reality, this asymmetric reproduction mechanism impairs the positive impact on cooperators through location synchronous effect on interdependent networks, which results in the adverse environment for cooperators.

In a recent work, Wu *et al*.^56^ found that the diversity of reproduction rate can enhance cooperation. The behavior of promoting cooperation is found to resemble coherence resonance. These findings suggest that the heterogeneity of individual traits might benefit cooperation in PD situation and give another clue to investigate the evolution of population between selfish individuals on interdependent networks. We hope our work can also inspire more studies for resolving the social dilemma, especially from the viewpoint of weight static networks and the individuals’ selection probability.

## Methods

**Figure 6:**
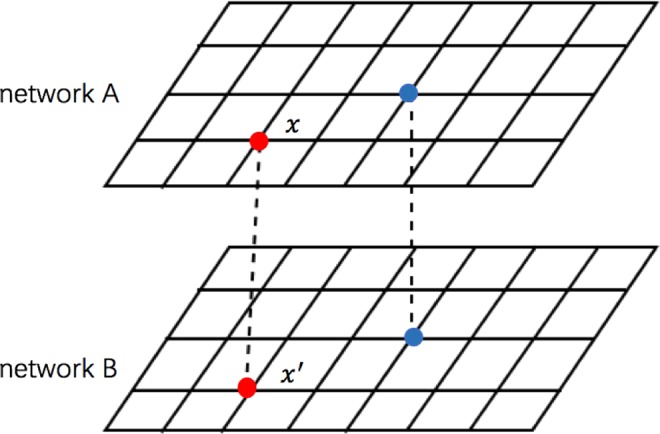
Schematic diagram of interdependent networks. We randomly show two different players in upper network (network A) and their partner in bottom network (network B). The two nodes are associated with each other.

We investigate the public goods game on interdependent networks (network *A* and network *B*), which are designed as *L* × *L* square lattices with periodic boundary condition, as shown in Fig. 6. Initially, each player on site *x* in network *A* and on site *x’* in network *B* is assigned either as a cooperator or a defector with equal probability. The calculation of payoffs *P*_*x*_ and *P*_*x*_ on both networks follows the same standard procedure. In detail, a cooperator contributes an amount *c* to the common pool (without loss of generality, we fix *c* = 1 in this article), while a defector contributes nothing. Then the sum of all contributions in each group is multiplied by an enhancement factor *r*, then the resulting amount is shared equally among all group members. We set the payoff player *x*(*x*′) obtained from each group is ${P}_{x}^{g}({P}_{{x}^{\text{'}}}^{g})$, that is

1 $$\{\begin{array}{ccc}{P}_{x}^{g}=\frac{r\,\ast \,{n}_{c}}{G}-1\, & if & {s}_{x}=C\\ {P}_{x}^{g}=\frac{r\,\ast \,{n}_{c}}{G}\, & if & {s}_{x}=D\end{array}$$

In addition, each player will participate in *G* = *k* + 1 groups of investments, where one group is a self-centered group, and other *k* groups are established around his *k* = 4 nearest neighbors, respectively. Thus, the total payoffs are ${P}_{x}=\sum _{g}{P}_{x}^{g}$ and ${P}_{x^{\prime} }=\sum _{g}{P}_{x^{\prime} }^{g}$.

Since these two networks don’t have physical connections, interdependencies are introduced through practical functions:

2 $$\{\begin{array}{rcl}{U}_{x} & = & \alpha {P}_{x}+(1-\alpha ){P}_{x^{\prime} }\\ {U}_{x^{\prime} } & = & (1-\alpha ){P}_{x^{\prime} }+\alpha {P}_{x}\end{array}$$

The asymmetric coupling method is considered into our model by introducing the parameter α^57^. The case of 0 < α ≤ 0.5 determines the degree of asymmetric coupling. In this paper, we only consider the influence of asymmetric reproductive capacity and avoid the bias caused by asymmetric coupling, we se*t* α = *0.5*. After determining the utility according to eq. (2), reproductive strategy is attempted between nearest neighbors on the given network. Thus, player *x*^′^ on network *B* can adopt the strategy ${s}_{y^{\prime} }$ from one of his randomly chosen nearest neighbors *y*^′^ with a probability determined by the Fermi function^58^:

3 $$W({s}_{y^{\prime} }\to {s}_{x^{\prime} })=\frac{1}{1+\exp [({U}_{x^{\prime} }-{U}_{y^{\prime} })/K]}$$

where utility ${U}_{y^{\prime} }$ of player *y*^′^ is calculated as same as ${U}_{x^{\prime} }$. Asymmetric reproductive capacity is introduced in the following pattern: players decide to update their strategy at each step on main network (network *B*), while, for sub network (network *A*), players update their strategies with a fixed probability *p*. Obviously, the system is restored to the traditional case when *p* = 1, where cooperation can survive by interdependent network reciprocity. In our system, asymmetric set-up is introduced when *p* < 1. It is worth stating that the process of strategy updating on the network *A* is determined if reproduction occurs. Without loss of generality, we set *K* = 0.5^59,60^ in Eq. (3).

Model simulations are performed through random sequential updates, each individual on two networks has an opportunity to renew strategy on average during a Monte Carlo step (MCS). The size of each network ranges from *L* = 400 to 1000 in order to avoid the effects of finite-size, and MCS required to 5 × 10^4^or 1 × 10^6^.
